# The Rourke Baby Record Infant/Child Maintenance Guide: do doctors use it, do they find it useful, and does using it improve their well-baby visit records?

**DOI:** 10.1186/1471-2296-10-28

**Published:** 2009-04-30

**Authors:** Leslie Rourke, Marshall Godwin, James Rourke, Sarah Pearce, Joyce Bean

**Affiliations:** 1 Discipline of Family Medicine, Faculty of Medicine, Memorial University of Newfoundland, St. John's, NL A1B 3V6, Canada; 2 Michael G. DeGroote School of Medicine Class of 2009, McMaster University, Hamilton ON, Canada; 3 Family practice nurse, Goderich, ON, Canada

## Abstract

**Background:**

The Rourke Baby Record (RBR) –  – is a freely available evidence-based structured form for child health surveillance from zero to five years. Family physicians/general practitioners (FP/GPs) doing office based well-baby care in three Ontario Canada cities (London, Ottawa, and Toronto) were randomly sampled to study the prevalence and utility of the RBR and documentation of well-baby visits.

**Methods:**

Database with telephone confirmation was conducted to assess the prevalence of use of the RBR.

Study Part 1: Questionnaire mailed to a random sample of 100 RBR users. Outcome measures were utility of, helpfulness of, and suggestions for the RBR. Descriptive analysis was employed.

Study Part 2: Retrospective chart review of well-baby visits by 38 FP/GPs using student t-tests and factor analysis. Outcome measures were well-baby visit documentation of growth, nutrition, safety issues, developmental milestones, physical examination, and overall comprehensiveness.

**Results:**

The RBR was used by 78.5% (402/512) of successfully contacted FP/GPs who did well-baby care in these 3 cities.

Study Part 1: Questionnaire respondents (N = 41/100) used the RBR in several ways, and found it most helpful for assessing healthy child development, charting/recording the visits, managing time effectively, addressing parent concerns, identifying health problems, and identifying high risk situations. The RBR was seen to be least helpful as a tool for managing or for referring identified health problems.

Study Part 2: Charts from a total of 1,378 well-baby visits on 176 children were audited. Well-baby care provided by the 20 FP/GPs who used the RBR compared to that by the 18 non-users was statistically more likely to include documentation of type of feeding (p = 0.023), discussion of safety issues (p < 0.001), assessment of development (p = 0.001), and overall comprehensiveness (p < 0.001). Well-baby care provided by the RBR users compared to that by the non-users was not more likely to include documentation of measurement of growth (p = 0.097), or physical examination (p = 0.828).

**Conclusion:**

The RBR was widely used by FP/GPs in these settings. RBR users found it helpful for many purposes, and had a consistently high rate of documentation of many aspects of well-baby care. The Rourke Baby Record has become a de facto gold standard clinical practice tool in knowledge translation for pediatric preventive medicine and health surveillance for primary care pediatric providers.

## Background

The Rourke Baby Record –  – is a freely available structured guide for family physicians/general practitioners (FP/GPs), paediatricians and others who provide well-baby/child care from zero to five years of age [1]. It was initially developed as a knowledge translation tool by Drs. Leslie and James Rourke in 1979 and first published in Canadian Family Physician in 1985 [2]. Over 30 years, the Rourke Baby Record (RBR) has evolved with revisions [3-8] to provide current, comprehensive evidence-based well-baby care. It is endorsed by both the College of Family Physicians of Canada (CFPC) [9] and the Canadian Paediatric Society (CPS) [10], and is cited in review articles and textbooks [11-15]. Grades of evidence of the individual items in the RBR are identified as good, fair, or consensus/no definitive evidence.

Research on structured forms to document well-baby/child care has generally found improved outcomes in documentation, parental satisfaction, and provider performance. Young found that FP/GPs using well-visit forms were significantly more likely to initiate anticipatory guidance discussions, to address concerns, and to provide handouts [16]. A structured encounter form resulted in significant improvement in documentation of most aspects of well-child care by family practice residents, including developmental assessment, safety and nutrition counseling, and measurement of growth, but not of physical examination [17]. In a study of pediatric house staff, structured encounter forms were associated with increased knowledge of developmental milestones and anticipatory guidance/preventive care, increased parent satisfaction, and improved compliance with recommended guidelines for developmental assessment [18]. Duggan found that use of a structured well-child visit form was associated with significantly higher levels of both documented and observed house staff performance [19]. The structured forms in these studies [17-19] were developed in-house, often based on American Academy of Pediatrics recommendations.

With its seemingly widespread use by family physicians in Canada, research on the RBR prevalence and utility as well as quality of well-baby visit documentation was needed. This is the first published study to assess the utilization of the Rourke Baby Record and the quality of documentation of well-baby visits by family physicians/general practitioners.

## Methods

**Figure 1:**
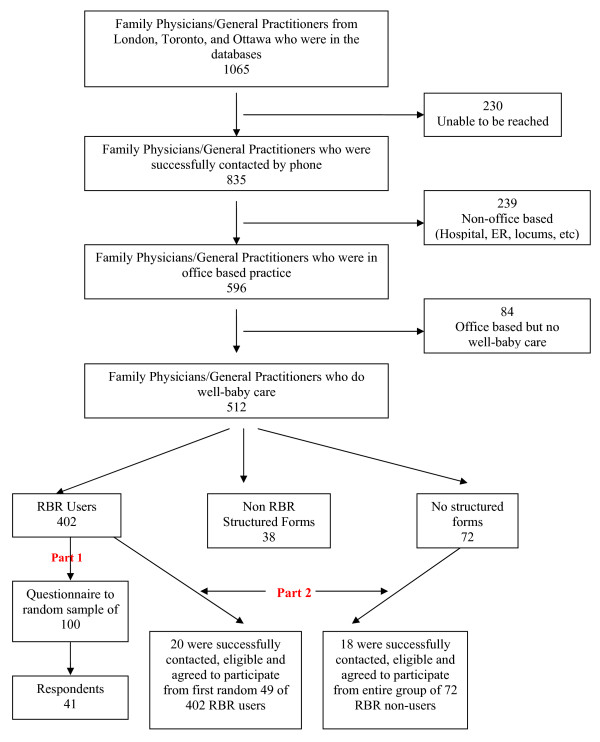
**Participant selection flow diagram**.

See Figure 1 for the participant selection flow diagram.

The research conformed to the Helsinki Declaration [20]. Ethics approval was obtained both from The University of Western Ontario Research Ethics Board for Health Sciences Research Involving Human Subjects (HSREB #11272E) and from the Human Investigation Committee (HIC #05.134) of the Faculty of Medicine at Memorial University of Newfoundland. Informed consent was obtained from all participating physicians. The questionnaire was piloted by several RBR-using FP/GPs, and the chart audit form was piloted both in practices who used and did not use the RBR.

### Rourke Baby Record prevalence of use

The 2005 Scott Medical Directory Database was used to identify family physicians and general practitioners who worked in three cities in Ontario, Canada: London, Toronto, and Ottawa. Contact information on these 1,065 physicians was crosschecked for accuracy with the College of Physicians and Surgeons of Ontario (CPSO) and Canada 411 online databases. Eight hundred and thirty-five of these were able to be successfully contacted by telephone in the spring of 2005 and brief verbal information obtained on their clinical activities. Of the 596 doing office-based practice, 512 were doing well-baby care in their practices and of these, 402 (78.5%) used the RBR as a clinical documentation tool, 38 (7.4%) used a variety of other structured forms, and 72 (14.1%) did not use a structured form for well-baby care.

### Study Part 1 – Rourke Baby Record Utility

To study the utility of the RBR, a random computer generated sample of 100 of the 402 RBR-using physicians was sent a questionnaire. It asked how the RBR was used, with six listed uses and comment space for others. The questionnaire then inquired about the helpfulness of the RBR. The physicians were asked to respond on a five point Likert scale (ranging from very unhelpful to very helpful) regarding ten aspects of well-baby care. The survey form also had two open-ended questions: one looking for other ways not previously listed that the RBR was particularly helpful; and a second asking for suggested improvements to the RBR. Descriptive analysis was done.

### Study Part 2 – Well-Baby Visit Documentation

Documentation of well-baby visits was studied through a retrospective chart audit. The outcome was a continuous variable based on a derived score from assessing multiple charts with multiple encounters (described later). Sample size was calculated based on the predicted differences in the chart audit scores for RBR users and non-users. The mean audit score was estimated to be 20 out of 25 items collected (predicted range 15 – 25) for RBR using physicians and 10 out of 25 (predicted range 5 – 15) for non-users. With a two tailed alpha of 0.05 and beta error of 0.20, a minimum of 16 physicians was required in each group [21]. The calculated minimum was exceeded, and this was slightly more in the user group.

Participants were obtained through the principal investigator contacting by telephone consecutive names on a random computer generated list of physicians in each group. To be eligible the physicians had to be able to be contacted within three attempts, they could not be using an electronic medical record or a structured well-baby visit record other than the RBR, they had to be in practice for longer than two years, and had to agree to have their charts audited by the research assistants. The sample of 20 RBR using physicians that were successfully contacted, were eligible and agreed to participate was derived from the first 49 on the randomly generated list of 402 RBR users. The sample of 18 non-RBR using physicians that were successfully contacted, were eligible and agreed to participate was derived from the full sample of 72 non RBR users.

No physicians received remuneration for participation, but were offered their individual and aggregate data for quality assurance purposes.

The study goal was to audit five randomly selected charts for each physician, of children aged 22 to 48 months at the time of the chart review. All well-baby visits in those charts up to and including the 18-month visit were included. The entire chart was examined to obtain data that could be found in different places – progress notes, growth charts, etc.

Six outcome variables reflecting documentation were assessed for each chart audited: growth, nutrition, safety issues, developmental milestones, physical examination, and overall comprehensiveness. The variables were defined as follows:

- **Growth**: all growth parameters recorded (height, weight, and head circumference);

- **Nutrition**: the type of feeding recorded;

- **Safety**: discussion of any safety issue(s) recorded;

- **Developmental Milestones**: any developmental milestones recorded;

- **Physical Examination**: a physical examination recorded.

The unit of analysis was the family physician. Each physician received a derived score on each of these variables based on the proportion of visits where the definition of each variable was met. (e.g.: the proportion of visits where all growth parameters were recorded, the proportion of visits where type of feeding was recorded, and so on). A sixth variable, **Overall Comprehensiveness**, was derived based on the mean proportion of the other five scores.

The scores in the RBR user group were compared with the scores in the non-user group using student t-tests. Factor analysis was used to test that the five items fit a single factor model of a comprehensive measurement of well-baby visits. Gender and date of birth were the only patient identifiers collected. Physician identifiers were removed when data analysis was complete.

## Results

### Study Part 1 – Rourke Baby Record Utility

Forty-one of the 100 RBR-using physicians who were sent the questionnaire responded. The 41 respondents were 51% male, they were all certified in family medicine through the CFPC, they had been in practice on average for 20 years, and 85% were in group practice rather than solo practice. Analysis of the responses provided an indication of how the RBR was utilized, the degree of satisfaction with the tool, and suggestions for RBR improvement.

The RBR was used primarily as a charting record (40/41 = 97.6%) and as an aide memoire for age appropriate evidence-based components of a well-baby/child visit (37/41 = 90%). Sixty-one percent reported use by other health care team members in the office. It was much less commonly used for communicating patient information to consultants (9/41 = 22%).

The value or helpfulness of the RBR to the doctors is shown in Table 1. It was seen as most helpful for assessing healthy child development, charting/recording the visits, managing time effectively, addressing parent concerns, identifying health problems, and identifying high risk situations such as safety issues and family problems. The RBR was seen to be least helpful for managing or for referring identified health problems. Other uses listed by respondents included: recalling guidelines, giving advice to parents, organizing the well baby visit, and use as a teaching tool.

**Table 1:** Study Part 1 – Helpfulness of the Rourke Baby Record (RBR).

	Somewhat or Very helpful N (%)
Assessing healthy child development	40/40 (100)

Recording/charting tool	37/39 (95)

Managing time effectively	36/40 (90)

Addressing parent concerns	34/40 (85)

Identifying health problems	32/40 (80)

Identifying high-risk situations (e.g. safety issues, family problems, etc.)	30/39 (77)

Charting growth parameters (ht, wt, HC)	29/40 (73)

Collaborating with other health care team members	25/40 (63)

Managing identified health problems	19/40 (48)

Referring identified health problems	14/39 (36)

Proportion of questionnaire respondents who felt that the RBR was somewhat or very helpful for the following purposes.

The most common suggestions for improvement to the RBR were: to expand the space for writing; to update and consolidate the immunization section; to improve growth charting; to expand the development section; and to make the RBR available in a well integrated electronic format.

### Study Part 2 – Well-baby Visit Documentation

To study documentation of well-baby visits, 176 patient charts were reviewed. These included 1,378 well-baby visits provided by the 38 participating FP/GPs – 20 of whom used the RBR and 18 of whom did not. The mean number of well-baby visit per child up to 22 months of age was 7.8 with a range from 1 to 13. An average of 4.6 charts per participating physician was reviewed.

The demographics of the 38 participating FP/GPs are shown in Table 2. The two groups (RBR users and non-users) were similar in terms of Canadian training, extra pediatrics training and involvement in some teaching. The only statistically significant difference between the groups was the number of years in practice. The RBR using physicians had been in practice for a shorter length of time.

**Table 2:** Study Part 2 – Demographics of the RBR using and non-using physicians

**Variable**		**Physicians who use the RBR** N = 20	**Physicians who do not use the RBR** N = 18
**Physician gender**	Male	6 (30%)	10 (55.6%)
	
	Female	14 (70%)	8 (44.4%)

Total years in practice*		16 Years (SD: 7.7)	26 years (SD:7.3)

	**London**	12 (60%)	8 (44.4%)
	
**Practice Location**	**Toronto**	5 (25%)	7 (38.9%)
	
	**Ottawa**	3 (15%)	3 (16.7%)

**Practice type**	**Solo**	3 (15%)	6 (33.3%)
	
	**Group**	17 (85%)	12 (66.7%)

**Remuneration**	**Fee-for-Service**	14 (70%)	10 (55.6%)
	
	**Other**	6 (30%)	8 (44.4%)

**Physician training**	**Fully Canadian Trained**	17 (85%)	16 (88.9%)
	
	**Foreign or Both**	3 (15%)	2 (11.1%)

**Involved in some teaching**		14 (70%)	12(66.7%)

**Extra paediatrics training**		2 (10%)	2 (11.1%)

*P < 0.001

Table 3 shows the results of the derived scores for documentation of well-baby visits in both RBR user and non-user groups. Well-baby care provided by RBR users compared to non-users was more likely to include documentation of type of feeding (p = 0.023), discussion of safety issues (p < 0.001), assessment of development (p = 0.001), and overall comprehensiveness (p < 0.001). Well-baby care provided by RBR users compared to non-users was not more likely to include documentation of measurement of growth (p = 0.097), and physical examination (p = 0.828).

**Table 3:** Study Part 2 – RBR using vs. non-using physicians: The proportion of well-baby visits where the documentation criteria for each of the six derived variables was met

**Outcome Variables**	**Physicians who used the RBR** **(Mean and 95% CI of their derived scores)**	**Physicians who did not use the RBR** **(Mean and 95% CI of their derived scores)**	**P Value**
***Growth*** (wt, length, and head circ recorded)	77.4% (95% CI: 64.9 – 89.9)	59.4% (95% CI: 41.3 – 77.6)	P = 0.097

***Nutrition*** (type of feeding recorded)	86.9% (95% CI: 79.9 – 93.9)	69.8% (95% CI: 56.6 – 83.0)	***P = 0.023***

***Safety*** (discussion of safety issue(s) recorded)	70.7% (95% CI: 57.9 – 83.5)	2.8% (95% CI: -0.4 – 6.0)	***P < 0.001***

***Development*** (any milestones recorded)	80.3% (95% CI: 72.3 – 88.4)	49.9% (95% CI: 35.7 – 64.1)	***P = 0.001***

***P/E*** (physical exam recorded)	89.4% (95% CI: 82.4 – 96.4)	88.1% (95% CI: 77.7 – 98.6)	P = 0.828

***Overall comprehensiveness*** (avg proportion estimate for each variable)	80.8% (95% CI: 74.3 – 87.3)	53.8% (95% CI:45.0 – 62.6)	***P < 0.001***

Factor analysis was used to test that the five items fit a single factor model of a comprehensive measurement of well-baby visits. Not only were all indicators in the same direction, but also each indicator was a satisfactory measure of the comprehensive scale. The overall reliability for the comprehensive scale was Alpha = .7535 and standardized item alpha = .7877, an excellent measure of reliability.

## Discussion

### Study Part 1 – Rourke Baby Record Utility

Almost 80% of FP/GPs in these three cities in Ontario who provide well-baby care use the RBR.

RBR users found the tool most useful for assessing child development. This is consistent with the literature which shows that a structured form improves provider performance and recording of developmental milestones [17-19]. FP/GPs performing well-baby care should be positioned to detect delays in healthy child development because of the sequential visits in the first years of life and because of their knowledge of the child in the context of the family and community.

More comprehensive care generally requires more time. The high reported value of the RBR to users as a charting/recording tool and for managing time effectively thus seem to suggest more comprehensiveness within the time available for the well-baby visit.

The least useful features of the tool were in managing and referring identified health problems. This was not unexpected as the RBR was not designed for these purposes. Subsequent to these findings, an attempt was made for the first time in the 2006 RBR [22] to incorporate a resource flow sheet for use when problems in development are found during the well-baby visit.

The variation in the strength of satisfaction with the RBR in different roles (from 36% somewhat or very helpful in referring identified health problems to 100% somewhat or very helpful in assessing healthy child development) also suggests that the respondents did not generalize their responses to all questions. This helps to validate the questionnaire as a true measure of these items.

In all surveys with a response rate less than 100%, it is important to know whether or not the non-respondents are different from the respondents. The demographics of the 41 RBR user questionnaire respondents were compared with those of the Canadian National Physician Survey (NPS) 2004 database of FP/GPs who practiced pediatrics in the same three cities (personal communication Sarah Scott July 18, 2007). This revealed a similar gender breakdown of 51% male in this study and 46.5% male in the NPS database. NPS data recorded mean FP/GP age of 48 years while the RBR questionnaire respondents had been in practice for a mean of 20 years, which correlates closely even though different demographics were measured. A lower proportion of RBR user questionnaire respondents were in solo practice (15%) than in the NPS database (30%). This could suggest that group practice with sharing of resources, ideas, updates, etc. may be associated with increased adoption of tools such as the RBR to enhance medical practice. A higher proportion of RBR user questionnaire respondents were involved in teaching (73%) than those in the NPS database (33%). The value of the RBR as a teaching tool was commented on by respondents. There was no comparable 2004 NPS data available for the remaining demographic items collected in the RBR user questionnaire. The response rate of 41% is comparable to the NPS CCFP FP/GP response rate of 42.8%.

### Study Part 2 – Well-baby Visit Documentation

In medicine there is a growing trend to more complete documentation of patient care. This has a primary goal of improved patient outcomes and also secondary goals of research analysis and system improvement. The literature described earlier showed that a structured form improves documentation and performance of well-baby visits [16-19].

This study highlights the Rourke Baby Record, an evidence-based structured form for well-baby/child visits from zero to five years of age. FP/GPs using the RBR demonstrated mean documentation scores for the five study variables ranging from 71 to 89% with a mean overall comprehensiveness score of 81%. RBR users compared to those not using the RBR had significantly better documentation of four key well-baby visit actions: discussion of safety issue(s), assessment of development, recording the type of feeding, and overall comprehensiveness. This concurs with the literature on improved well-baby visit documentation with structured forms. The RBR –  – is a freely available tool easily incorporated into primary care paediatric practice.

There are several limitations to this study. This research was not designed to study parent perceptions of or satisfaction with the well-baby visits. This could be an area for further research.

The widespread use of the RBR (78.5% in these settings) makes it difficult to compare the vast majority (RBR users) with a small minority of outliers (RBR non-users) as other variables cannot be excluded. For example, the older age of the RBR non-users could be a confounding variable.

Documentation is an indirect measurement of well-baby care. Studies comparing performance and recording of well-baby visits have shown that the use of a structured form is associated with significantly higher levels of both recorded and observed performance [18,19]. Comparison of the actual content of the visits would require a prospective study with observation and/or recording of the visits and/or parent recollection at the end of the visits. The same issue of comparing a majority using a well-accepted tool with a minority of non-users would also apply in this situation.

A prospective RCT study assessing the value of the RBR in patient health outcomes such as detecting and managing abnormalities found during the well-baby visit, or in preventing injuries or illness, would require huge resources and would be ethically questionable given the current widespread use of the RBR. It would also have to occur in a setting where the RBR is not widely used.

The Rourke Baby Record could be used in future research as a data collection tool to study baseline data and outcomes including the predictors of, barriers to, and impact of optimal well-baby care.

## Conclusion

This study found that the large majority of family physicians doing well-baby care in three cities in Ontario Canada use a freely available evidence-based structured form, the Rourke Baby Record –  – for well-baby visits. Users described the RBR as most useful for assessing child development, for recording the well baby visit, for managing time effectively, for addressing parent concerns and for identifying health problems and high-risk situations.

Well-baby visit documentation by FP/GPs using the RBR was comprehensive for all studied variables including not only physical examination and measurement of growth, but also was statistically more likely to include type of feeding, discussion of safety issues, assessment of development, and overall comprehensiveness.

Our study demonstrates that the Rourke Baby Record is a valuable knowledge translation tool for well-baby care. It is however, only an aid to guide the listening, flexibility, and communication skills required for compassionate patient-centred care.

## Competing interests

The authors declare that they have no competing interests.

## Authors' contributions

LR was involved in all parts of the study. MG was involved in data analysis and interpretation and in critical revision of the article. JR was involved in the conception and study design, data interpretation and critical revision of the article. SP and JB were involved in data acquisition, entry and analysis. All authors read and approved the final manuscript.

## Authors' information

Leslie Rourke, MD, CCFP, MClinSc, FCFP is an Associate Professor of Family Medicine at Memorial University of Newfoundland, Canada and a former rural family physician in Goderich, Ontario, Canada.

Marshall Godwin, MD, MSc, CCFP, FCFP is Director of the Primary Healthcare Research Unit and Professor of Family Medicine at Memorial University of Newfoundland, Canada.

James Rourke, MD, CCFP(EM), MClinSc, FCFP is Professor of Family Medicine and Dean of Medicine at Memorial University of Newfoundland. Previously he was Assistant Dean, Rural and Regional Medicine at The University of Western Ontario and a family physician in Goderich, Ontario.

Sarah Pearce is a medical student graduating in 2009 from the Michael G. DeGroote School of Medicine at McMaster University, Canada.

Joyce Bean, RN, has worked in health care in rural hospitals, family practice clinics, and a youth detention centre in Ontario.

## Pre-publication history

The pre-publication history for this paper can be accessed here:


